# Mechanism of ultrasonic-alkali-thermal modification for enhancing the emulsifying properties of rice protein and its stability in high-internal-phase emulsions

**DOI:** 10.1016/j.ultsonch.2025.107469

**Published:** 2025-07-18

**Authors:** Lijie Zhu, Haiqiang Liao, Kun Zhuang, Shangyuan Sang, Lei Chen, Xianhui Chang, Qi Zhang, Qingyun Lv, Xiuying Liu, Xinqi Liu, Wenping Ding

**Affiliations:** a Key Laboratory for Deep Processing of Major Grain and Oil, Ministry of Education, School of Food Science and Engineering, Wuhan Polytechnic University, Wuhan, Hubei 430028, China; b China Food Flavor and Nutrition Health Innovation Center, Beijing Technology & Business University, Beijing 100048, China; c College of Food Science and Engineering, Ningbo University, Zhejiang, Ningbo 315211, China

**Keywords:** Rice protein, Ultrasound, Alkali-thermal, Emulsification property, High-internal-phase emulsion

## Abstract

Rice protein, as a hypoallergenic plant protein, faces limitations in food industry applications due to the poor emulsification of its native form. In this study, rice protein was subjected to ultrasonic, alkali-thermal, and combined ultrasonic-alkali-thermal treatments. Experimental results revealed that the ultrasonic-alkali-thermal combined treatment significantly reduced the particle size to 129.67 nm. Structural analysis showed an increase in random coil content within the secondary structure, indicating a transition from ordered to disordered conformation. Contact angle measurements demonstrated improved intermediate wettability of the modified protein. The high-internal-phase emulsion prepared with ultrasonic-alkali-thermal treated rice protein exhibited enhanced stability, with the Turbiscan Stability Index as low as 0.39, reduced emulsion particle size (14.17 μm), and elevated storage moduli. These findings collectively suggest that the ultrasonic-alkali-thermal treatment is an effective strategy for enhancing the emulsifying properties of rice protein.

## Highlights

• Ultrasonic-alkali-thermal enhanced the surface hydrophobicity of rice protein.
• Assess interface adsorption by measuring interfacial tension and contact angle.
• Ultrasonic-alkali-thermal treated rice protein decreased emulsion particle size.
• Ultrasonic-alkali-thermal enhanced the ability of rice protein to stabilize emulsion.

## 1 Introduction

The country with the largest rice production in the world is China. According to the statistics of the National Bureau of Statistics of China, China's rice production in 2024 reached 207.535 million tons [1]. In the process of finishing rice into rice, rice husk, rice bran, rice embryo and broken rice will be produced, etc [2]. These by-products contain a large amount of protein that is not fully utilized, resulting in a huge waste of protein resources [3]. Protein is the second most abundant component in rice, accounting for about 8 % of the mass of rice, and the proteins from rice are collectively referred to as rice protein. Rice protein has many advantages compared with other plant proteins, for example, most plant proteins contain anti-nutritional factors, soy protein mostly contains protease inhibitors and phytohemagglutinin [4], and wheat protein contains gluten, which is not conducive to people with gluten allergy [5], while rice protein does not contain these substances. Rice protein is free from anti-nutritional factors and gluten, making it suitable for infants, young children, and people with gluten intolerance [6,7]. Furthermore, it has potential functions such as lowering cholesterol, exhibiting antioxidant activity, and regulating blood lipids [8,9].

Although rice protein has many advantages, it is very limited in industrial application due to its own nature. Rice protein is mainly composed of glutelin, and the rest are salt-soluble protein, water-soluble protein and alcohol-soluble protein. Glutelin intramolecular and intermolecular cross-linking through disulfide bonds forms an anti-parallel β-sheet conformation, resulting in high surface hydrophobicity and extremely low solubility (usually less than 5 %) under neutral conditions [10]. Therefore, the emulsifying ability, foaming capacity, and stability of natural rice protein under neutral conditions are not ideal, limiting its application as an emulsifier in such conditions.

In order to change this situation, most scholars increase the hydrophilic groups of rice proteins by phosphorylation [11], glycosylation [12] and other methods, or use various proteases [13] to hydrolyze the hydrophobic regions of proteins in order to improve the exposure of hydrophilic groups. While such chemical or biological modification methods enhance the solubility, emulsifying capacity, and emulsion stability of proteins, they compromise nutritional value and may introduce chemical residues or generate toxic peptides. In contrast, physical modification methods (ultrasonic treatment, thermal treatment, etc.) have better safety and economic benefits [14]. Rice glutelin undergoes unfolding and subunit dissociation under alkaline conditions. At this point, when other pretreatment methods are applied to the protein, it is more likely to damage its internal structure[14]. Heat treatment is a commonly used physical modification method. It can break disulfide bonds in proteins, induce structural transitions, and enhance molecular flexibility, thereby enabling better adsorption at interfaces[14]. Ultrasonic processing technology has the characteristics of high efficiency, energy saving and environmental protection, and is used in protein processing to improve the solubility of proteins and change the functional properties of proteins[15,16]. As a non-thermal treatment, ultrasound has cavitation effect and mechanical effect. Cavitation destroys the quaternary structure of proteins, generates small molecular subunits and increases the solubility of proteins, mechanical effects can uniformly disperse samples and crush large solid particles [17]. The modification effect can be improved by assisting other methods in the process of ultrasound.

The modified rice protein has improved amphiphilicity and is usually used as a emulgator for emulsions. In existing studies, rice protein is mostly used to stabilize low internal phase emulsions and Moderate internal phase emulsion (dispersed phase volume fraction < 74 %), which have low viscosity and are prone to droplet aggregation and emulsion breaking during storage [18]. The high-internal-phase emulsion (dispersed phase volume fraction > 74 %) has high viscosity, good viscoelasticity and deformation resistance, and usually shows good stability during storage. At the same time, the solid-like properties and shear-thinning properties of the high-internal-phase emulsion itself make it widely used in fat substitutes, food 3D printing inks and other fields[19]. Nevertheless, research on using rice protein to stabilize high-internal-phase emulsions remains limited.

In this experiment, rice protein was modified by three methods: alkali-thermal, alkali-ultrasound, and alkali-ultrasound-thermal treatment to study the advantages of combined treatment compared with single treatment. The changes in its structure were analyzed through spectral analysis and electrophoresis results, and the modified sample was prepared into high-internal-phase emulsion. The emulsion size, microstructure and storage stability were used to determine whether the emulsification performance of modified rice protein was improved. It is hoped that the utilization of rice protein can be expanded.

## 2 Methods

### 2.1 Materials

Rice and rice bran oil were supplied by Yihai Jiali Golden Arowana Food Group Co., LTD, Wu Han, China. Bicinchoninic Acid (BCA) kits were purchased from Source Leaf Biotechnology Co., LTD, Wu Han, China. The reagents employed were of analytical grade and commercially available. Deionized water was prepared using a pure water system (ECO-Q15YT, HHItrch Instrument Co., LTD, Shang Hai, China).

### 2.2 Methods

#### 2.2.1 Extraction of rice protein

The separation method of rice protein was slightly modified according to the method of Huang el at[20]. The rice was crushed by a hammer cyclone mill through a 100-mesh sieve. The rice flour and deionized water were prepared at a solid-to-liquid ratio of 1:10 (w/v). The pH of the suspension was adjusted to 11 using a NaOH solution, followed by heating in a 40℃ water bath and subjected to magnetic stirring for 4 h. Subsequently, the suspension was centrifuged at 4000 rpm for 20 min to obtain the supernatant. The pH of the supernatant was adjusted to 4.5, followed by centrifugation at 4000 rpm for 20 min to obtain the precipitate. The precipitate was washed three times with deionized water and freeze-dried. The protein content was determined to be 90.02 % using the Kjeldahl nitrogen determination method.

#### 2.2.2 Preparation of ultrasound- alkali-thermal modified rice protein

Prepare six 100 mL protein suspensions at 4 % (w/v) using deionized water. Adjust the pH to 7 and hydrate for 30 min. The pH of the protein suspension was then adjusted to 12, and the following treatments were performed:

(1) The control group was treated with no treatment, (2) 50 ℃ water bath for 2 h, (3) 200 W ultrasound for 20 min, (4) 600 W ultrasound for 20 min, (5) 200 W ultrasound for 20 min, and then 50 ℃ water bath for 2 h, (6) 600 W ultrasound for 20 min, and then it was treated in a water bath at 50 ℃ for 2 h. All samples were first cooled in an ice-water bath (0–4 °C), then adjusted to pH 7 through neutralization with 0.1 M HCl. The samples were named Rice protein (RP), Alkali-thermal treatment (AH), 200 W ultrasonic treatment (U2), 600 W ultrasonic treatment (U6), 200 W ultrasonic-alkali-thermal treatment (UAH2), 600 W ultrasonic-alkali-thermal treatment (UAH6), freeze-dried for use.

#### 2.2.3 Solubility

Weigh and dissolve a 10 mg sample in 10 mL of phosphate buffer (0.01 mol/L, pH 8). Stir with a magnetic force stirrer at room temperature for 2 h, then centrifuge at 3000 × g for 15 min. Take the supernatant and use a BCA kit to determine the protein content in the supernatant. The principle of the BCA method is based on the reduction of bivalent copper ions to cuprous ions by peptide bonds. These cuprous ions then form a complex with BCA under alkaline conditions to produce a purple-red complex. The absorbance is measured, and a standard curve is plotted to calculate the protein content. The concentrations of the standard curves are 0, 0.05, 0.1, 0.15, 0.2, 0.3, 0.4, and 0.5 mg/mL. The solubility is calculated using the following formula:

(1) $$\text{Solubility =}\;\frac{\text{Protein content of supernatant}}{\text{Protein content in the sample}}\times \text{100\%}$$

#### 2.2.4 Emulsification performance

Prepare a protein dispersion at a concentration of 10 mg/mL. Mix 15 mL of the dispersion with 5 mL of rice bran oil and homogenize at 10,000 rpm for 2 min. Immediately thereafter, withdraw 50 µL of the sample from the bottom layer and add it to 5 mL of 0.1 % SDS solution, followed by vortexing for 30 s. Measure the absorbance (A0) at 500 nm using a UV spectrophotometer. After allowing the mixture to stand for 10 min, repeat the aforementioned procedure and record the absorbance as A10. Use a 0.1 % SDS solution as the blank. The calculation formula is as follows:

(2) $$\text{EAI}\left({\text{m}}^{\text{2}}/\text{g}\right)=\frac{\text{2}\times \text{2.303}\times {\text{A}}_{\text{0}}\times \text{N}}{\text{C}\;\times \;\varphi \;\times \;{\text{10}}^{\text{4}}}$$

(3) $$\text{ESI}\left(\text{\%}\right)=\frac{{\text{A}}_{\text{10}}}{{\text{A}}_{\text{0}}}\times \text{100\%}$$

In the formula: *N* represents the dilution factor, *C* represents the protein concentration, and *φ* represents the volume fraction of the aqueous phase.

#### 2.2.5 Zeta potential and particle size

Prepare a protein dispersion at a concentration of 1 mg/mL using deionized water. Adjust the pH to 7 and stir at room temperature for 2 h. Then dilute it with deionized water to obtain a protein dispersion of 0.1 mg/mL. Transfer it to the sample cell and analyze it using a Malvern particle size analyzer (Zetasizer Nano ZS, Malvern Instruments LTD., UK). The parameters are set as follows: Protein refractive index: 1.45, water refractive index: 1.33.

#### 2.2.6 Scanning electron microscope (SEM)

Place a small amount of sample powder onto the double-sided conductive adhesive, adhere the conductive adhesive to the sample plate, and gently blow away excess powder and remove any sample outside the conductive adhesive using an ear swab. Then sputter-coat with gold for 120 s. Observe the surface structure of the sample at an accelerating voltage of 20 kV using a scanning electron microscope (SU8600, Hitachi, Japan).

#### 2.2.7 Endogenous fluorescence spectroscopy

The sample was prepared into a 0.1 mg/mL protein dispersion using phosphate buffer (0.01 mol/L, pH8). The fluorescence spectra were measured using a fluorescence spectrophotometer (F-4600, Hitachi, Japan). The excitation wavelength was 290 nm, the emission wavelength range was 300–500 nm, the slit width was 2.5 nm, the acceleration voltage was 700 V, and the response time was set to 0.5 s.

#### 2.2.8 Surface hydrophobicity

The sample was prepared into a protein dispersion of 0.1 mg/mL using phosphate buffer (0.01 mol/L, pH8), and then diluted into dispersions of 0.02, 0.04, 0.06, 0.08, and 0.1 mg/mL. ANS was used as the fluorescence probe. Take 4 mL of the protein dispersion and mix it evenly with 20 µL of freshly prepared ANS solution (8 mmol/L). The fluorescence intensity of the mixture was measured using a fluorescence spectrophotometer (F-4600, Hitachi, Japan) at an excitation wavelength of 390 nm and an emission wavelength of 300–500 nm. The slit width was 2.5 nm, the acceleration voltage was 700 V, and the response time was set to 0.5 s. The maximum fluorescence intensity (F1) was recorded.

Surface hydrophobicity is expressed as the initial slope of the plot of fluorescence intensity (F1) versus protein concentration, calculated through linear regression analysis.

#### 2.2.9 Ultraviolet spectrum

Prepare a protein dispersion at a concentration of 0.5 mg/mL using phosphate buffer (0.01 mol/L, pH 8). Then dilute it to 0.1 mg/mL and scan the spectrum between 200–400 nm using a UV–vis spectrophotometer (Evolution 220, Thermo Electron, USA). Determine the ultraviolet absorption spectrum of the sample and perform baseline correction using the solvent as the reference.

#### 2.2.10 SDS-polyacrylamide gel electrophoresis (SDS-PAGE)

The composition of the samples was studied by SDS-PAGE using 5 % concentrated gel and 12 % separation gel (Zhi et al., 2022). Dissolve the sample by mixing it with Tris-Glycine SDS buffer at a volume ratio of 4:1 and containing β-mercaptoethanol reducing agent. Heat it in a boiling water bath for 10 min, then centrifuge it at 10,000 × g for 10 min. Subsequently, add the supernatant (10 μL) and protein ladder (10 μL) to the pre-prepared gel for electrophoresis. The experimental conditions were set at 80 V for 30 min and 120 V for 1 h. Stain the gel with a solution containing 0.1 % Coomassie bright blue (R-250) for 30 min. Then destain with deionized water until the bands are clear. Gel images were observed using the chemiluminescence gel imaging system (ChemiDoc MP, Bole Corporation, USA).

#### 2.2.11 Interfacial tension

Prepare a protein dispersion at a concentration of 0.1 % using deionized water. The dynamic interfacial tension of different protein samples at the oil–water interface was determined employing the pendant drop technique via an interfacial tensiometer (DSA30S, KRUSS, Germany). A specific volume was drawn using a microinjection needle. The height of the sample stage was adjusted, and the needle was inserted into the quartz tank containing rice bran oil to release approximately 45 μL of the protein solution. Images of the droplet during the 3,600-second static period were continuously collected using the high-speed camera system and digitally processed. Then, the interfacial tension of the droplet was calculated by fitting the data to the Young-Laplace equation.

#### 2.2.12 Contact angle

Contact angle measurements of various protein specimens were conducted through the static sessile drop technique utilizing an interfacial tension meter. Deposit a drop of the protein dispersion onto a slide and allow it to air dry to form a film. Repeat this process three times. Place the slide on the sample stage, draw deionized water into the syringe, and release 3 μL of liquid droplets. Continuously collect images of the liquid droplets during the 10-second static period using the high-speed camera system and perform digital processing. Then, fit the contact angle data to the Young-Laplace equation.

#### 2.2.13 Secondary structure

Place the protein solution at a concentration of 0.1 mg/mL into the circular dichroism (CD) spectral polarizer and perform scanning between 190–260 nm. The scanning speed is 100 nm/min, the slit width is 1 nm, and the cuvette width is 1 mm. The secondary structure was analyzed using the CDNN software.

#### 2.2.14 Preparation of High-Internal-Phase emulsions

Different protein samples were prepared as protein dispersions at a concentration of 4 % (w/v) using deionized water. Hydrate the dispersions in a refrigerator at 4℃ overnight. Take 12.5 mL of the protein dispersion and add it to 37.5 mL of rice bran oil in a beaker. Disperse them using a high-speed disperser at 13,000 rpm for 30 s, followed by dispersion using an egg beater for an additional 30 s to obtain an emulsion with an oil phase concentration of 75 %.

#### 2.2.15 Emulsion potential

Take 1 mL of the emulsion and dilute it 20 times with deionized water. Transfer it to the sample cell and analyze it using a Malvern particle size analyzer.

#### 2.2.16 Emulsion particle size

The droplet sizes (D_43_) and particle size distribution of fresh emulsions were tested using a laser particle size analyzer (Master sizer 3000, Malvern Corporation, USA). The parameters are set as follows: Particle refractive index: 1.47, medium refractive index: 1.33.

#### 2.2.17 Confocal laser scanning microscope (CLSM)

The morphology of fresh emulsion was observed using a CLSM (OLYMPUS FV1200, Olympus Corporation, Japan). Take 1 mL of fresh emulsion, add 20 μL of Fluorescein Isothiocyanate (FITC) staining solution and 20 μL of Nile red staining solution, mix well, and then drop it onto a clean slide. Gently cover it with a coverslip to prevent the formation of air bubbles. Observe the samples at excitation wavelengths of 530 nm (Nile red) and 498 nm (FITC).

#### 2.2.18 Optical microscope

Take 10 μL of fresh emulsion, deposit it onto a clean slide, and gently cover it with a coverslip to prevent the formation of air bubbles. The sample was observed using an optical microscope (SDPTOP, Ningbo Sunny Optical Instrument Co., LTD., China) at 100 × magnification.

#### 2.2.19 Storage stability

The short-term stability of the prepared emulsion was analyzed using a multiple light scattering analyzer (Lab expert, Formulaction Company, France). During the experiment, 28 mL of the sample was accurately drawn and added to a dedicated multi-light glass test bottle, after which the scanning was initiated (one scan every 2 min for a total of 1 h). This instrument performs static vertical scanning of the sample. After multiple scans, a scattering spectrum capable of analyzing the emulsion state is obtained. The Turbiscan Stability Index (TSI) of the sample was calculated by analyzing the transmitted light and scattered light data using the system software.

#### 2.2.20 Data processing and analysis

All data were obtained from three parallel tests, with the average values recorded and the standard errors computed. The final results were expressed as the mean ± standard error. Univariate ANOVA analysis was performed using SPSS 22.0, with the Duncan test conducted for multiple comparisons. Different letters between the results denote statistically significant differences (p < 0.05). The graph was constructed using Origin software.

## 3 Results and discussion

### 3.1 Potential and particle size

**Fig. 1:**
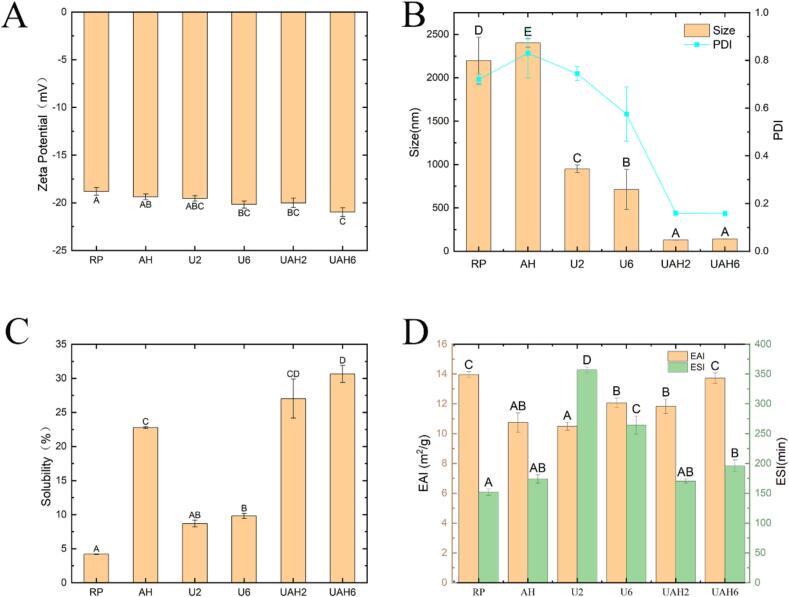
(A) The influence of different modification methods on the zeta potential of rice protein. (B) The influence of different modification methods on the particle size of rice protein. (C) The influence of different modification methods on the solubility of rice protein. (D) The influence of different modification methods on the emulsifying performance of rice protein. Note: Different letters between the results denote statistically significant differences (p < 0.05). Rice protein (RP), Alkali-thermal treatment (AH), 200 W ultrasonic treatment (U2), 600 W ultrasonic treatment (U6), 200 W ultrasonic-alkali-thermal treatment (UAH2), 600 W ultrasonic-alkali-thermal treatment (UAH6).

Fig. 1A shows the zeta potential of rice protein before and after modification. The zeta potential of RP was −18.8 ± 0.40 mV. After heat treatment, the zeta potential decreased to −19.37 ± 0.29 mV (AH), indicating that heat treatment exposed charged groups and altered the protein's spatial structure. Ultrasonic treatment also reduces the zeta potential of rice proteint. As the ultrasonic power increases, the zeta potential decreases from −19.53 ± 0.30 mV in U2 to −20.17 ± 0.37 mV in U6. The zeta potential of rice protein treated with ultrasonic-alkali-thermal treatment was the lowest at −20.97 ± 0.49 mV (UAH6). This may be because charged groups were initially exposed by ultrasonic treatment and further exposed during heat treatment. Although the zeta potential decreased across all treatments, no marked difference was observed between different treatment modalities. Fig. 1B shows the particle size of rice protein. The particle size of AH increased to 2402.33 ± 47.79 nm, indicating protein aggregation during heat treatment. The particle size of the ultrasonic treatment groups (U2 and U6) decreased to 948.47 ± 44.80 nm and 713.13 ± 232.63 nm, respectively. This may be due to the cavitation effect of ultrasound dispersing aggregated proteins. The particle sizes of the ultrasonic-alkali-thermal groups (UAH2 and UAH6) were the smallest at 129.67 ± 0.67 nm and 142.07 ± 0.35 nm, respectively. During heat treatment, the charged groups exposed by ultrasound caused electrostatic repulsion between proteins, preventing thermal aggregation[21]. Zhi et al. [22] also reached similar conclusions through ultrasound and heat treatment of pea protein.

### 3.2 Solubility

The solubility of rice protein is an important factor that limits its application in the food industry and affects its functional properties[23]. As shown in Fig. 1C, the solubility of RP is relatively low, at only 4.19 ± 0.03 %. This is because RP contains a large amount of glutelin. The hydrophobic amino acid residues within glutelin are interrelated, forming a tight protein network that limits the solubility of rice protein. The solubilities of U2 and U6 were 8.68 ± 0.50 % and 9.81 ± 0.37 %, respectively, representing a 107 % increase in solubility due to ultrasound treatment. The solubility of AH is 22.78 %, representing a 444 % increase compared with RP. The solubilities of UAH2 and UAH6 were 27.26 ± 2.87 % and 30.64 ± 1.25 %, respectively, representing increases of 545 % and 631 % compared to RP. The combined treatment and modification resulted in the greatest improvement in solubility. This may be because under alkaline conditions, the originally compact protein structure unfolds [24]. The cavitation effect of ultrasound disrupts the hydrophobic interactions between proteins and reduces the particle size of protein aggregates[25]. When heat-treated at 55 °C, the protein transforms from a rigid sphere to a molten sphere. Further exposure of the hydrophilic groups previously wrapped inside enhances the protein's solubility [26].

### 3.3 Emulsification

The emulsifying performance of proteins reflects their adsorption capacity at the oil–water interface and the anti-deformation ability of the formed emulsion. Emulsion stability index (ESI) indicates a protein's emulsion-stabilizing ability, whereas emulsification activity index (EAI) reflects its emulsifying capability. As shown in Fig. 1D, the emulsifying activity of the treatment groups decreased to varying degrees compared to RP (EAI: 13.95 ± 0.21 m^2^/g), while emulsifying stability showed varying degrees of improvement compared to RP (ESI: 151.77 ± 5.17 min). Additionally, the EAI of the protein exhibited ultrasonic power dependence. The EAI of U6 (12.06 ± 0.34 m^2^/g) was higher than that of U2 (10.50 ± 0.26 m^2^/g), and the EAI of UAH6 (13.73 ± 0.35 m^2^/g) was higher than that of UAH2 (11.83 ± 0.49 m^2^/g). This may be because the aggregation effect of heat treatment and the cavitation effect of ultrasonic treatment disrupt the original hydrophilic-hydrophobic balance of rice protein, thereby reducing emulsifying activity and enhancing emulsifying stability[27].

### 3.4 Ultraviolet spectrum

**Fig. 2:**
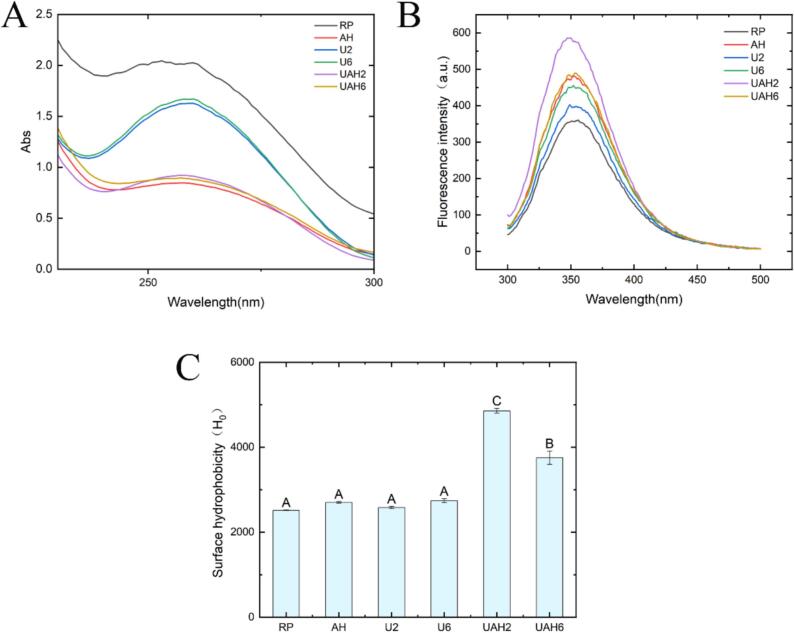
(A) Ultraviolet spectra of different modified rice proteins. (B) Fluorescence spectra of different modified rice proteins. (C) Effects of different modification methods on the surface hydrophobicity of rice proteins. Note: Different letters between the results denote statistically significant differences (p < 0.05). Rice protein (RP), Alkali-thermal treatment (AH), 200 W ultrasonic treatment (U2), 600 W ultrasonic treatment (U6), 200 W ultrasonic-alkali-thermal treatment (UAH2), 600 W ultrasonic-alkali-thermal treatment (UAH6).

Ultraviolet spectroscopy mainly reflects changes in the tertiary structure of proteins and the absorption intensity of the hydrophobic aromatic amino acid phenylalanine [28]. As shown in Fig. 2A, the ultraviolet absorption intensity of all treatment groups was lower than that of RP. Among these, the absorption peak of AH was the lowest, followed by UAH2 and UAH6, whereas the absorption peaks of U2 and U6 were much higher than those of the former three groups. These results indicate that both heat treatment and ultrasonic treatment affect the structure of rice protein, but heat treatment has a more significant effect than ultrasonic treatment. The reduction in the absorption peak may be attributed to changes in the protein's hydrophobic regions during heat and ultrasonic treatments, causing previously exposed phenylalanine residues to become embedded. Zhang et al. [29] also reported a decrease in the ultraviolet absorption peak of corn alcohol-soluble protein after heat treatment.

### 3.5 Fluorescence spectrum

The endogenous fluorescence spectrum of rice protein, measured at an excitation wavelength of 290 nm, primarily originates from specific tryptophan residues. Fluorescence changes can be used to observe alterations in the tertiary structure of the protein, thereby revealing its conformation and folding state [30]. As shown in Fig. 2B, different treatments cause structural changes in rice protein to varying degrees. Among these, UAH2 exhibits the highest fluorescence intensity, followed by UAH6, and both show a blue shift in their maximum emission wavelengths. These results indicate that the protein undergoes folding during processing, exposing tryptophan to a hydrophilic environment[30]. The lower fluorescence intensity of UAH6 compared to UAH2 may be attributed to fluorescence quenching caused by high-power ultrasound treatment [31].

### 3.6 Surface hydrophobicity

The surface hydrophobicity of proteins can reflect their solubility, emulsification properties, and structural changes. The content of hydrophobic groups can be assessed using ANS fluorescent probes. As shown in Fig. 2C, the surface hydrophobicity of the UAH2 and UAH6 groups is much higher than that of the other four groups. The surface hydrophobicity of the AH, U2, and U6 groups, while not significantly different from that of RP, is still higher than that of RP. These results indicate that ultrasonic-alkali-thermal treatment significantly enhances protein surface hydrophobicity, exposing the protein's hydrophobic groups to the environment. Both surface hydrophobicity and fluorescence spectroscopy results indicate that hydrophobic groups in treated rice protein are exposed, with ultrasonic-alkali-thermal treatment showing the best effect.

### 3.7 Secondary structure

CD spectroscopy is a common method for analyzing the secondary structure of proteins. By analyzing the CD spectrum, the content of the secondary structure can be obtained, as shown in Table 1. After ultrasonic treatment, the α-helix content of U2 and U6 increased to 7.19 ± 0.10 % and 6.72 ± 0.10 %, respectively, and the β-sheet content increased to 26.47 ± 0.05 % and 25.50 ± 0.35 %, respectively. The ordered structure increased, whereas the contents of β-turn and irregular coil decreased, indicating that ultrasonic treatment transformed the secondary structure of rice protein from disordered to ordered. Hong et al. [32] also found that ultrasound can make the structure of proteins more ordered. After heat treatment, the α-helix content in rice protein decreased from 6.51 % ±0.05 %to 5.22 ± 0.06 %, the β-turn content decreased to 27.73 ± 0.07 %, and the irregular coil content increased to 47.08 ± 0.08 %. The protein as a whole transformed into a more disordered structure. Long et al. [33] analyzed the rice residue protein after thermal denaturation and found that it had a more loosely structured and disordered conformation. The secondary structure changes in the combined treatment groups (UAH2 and UAH6) were similar to those observed after heat treatment, with an overall tendency toward a disordered structure. This might be due to the combined treatment involving ultrasonic treatment followed by heat treatment. It is worth noting that the changes in β-sheet were similar to those observed with ultrasonic treatment, indicating that β-sheet has good thermal stability. Studies have shown that when the disordered structure of a protein increases, the protein becomes looser, exposing more groups and thereby enhancing its functionality [34].

**Table 1:** Effects of Different Modification Methods on the secondary structure of rice protein.

	α-helix	β-sheet	β-turn	Random coil
RP	6.51 ± 0.05 %^B^	19.46 ± 0.69 %^A^	29.03 ± 0.23 %^D^	45.01 ± 0.5 %^BC^
AH	5.22 ± 0.06 %^A^	19.98 ± 0.04 %^AB^	27.73 ± 0.07 %^C^	47.08 ± 0.08 %^D^
U2	7.19 ± 0.10 %^C^	26.47 ± 0.05 %^C^	26.22 ± 0.11 %^B^	40.12 ± 0.05 %^A^
U6	6.72 ± 0.10 %^B^	25.50 ± 0.35 %^C^	26.52 ± 0.17 %^B^	41.27 ± 0.28 %^A^
UAH2	5.40 ± 0.10 %^A^	25.48 ± 0.64 %^C^	25.48 ± 0.09 %^A^	43.65 ± 0.64 %^B^
UAH6	5.20 ± 0.11 %^A^	21.72 ± 1.16 %^B^	26.81 ± 0.41 %^B^	46.29 ± 0.86 %^C^

Note: Different letters between the results denote statistically significant differences (p < 0.05). Rice protein (RP), Alkali-thermal treatment (AH), 200 W ultrasonic treatment (U2), 600 W ultrasonic treatment (U6), 200 W ultrasonic-alkali-thermal treatment (UAH2), 600 W ultrasonic-alkali-thermal treatment (UAH6).

### 3.8 Interface properties

**Fig. 3:**
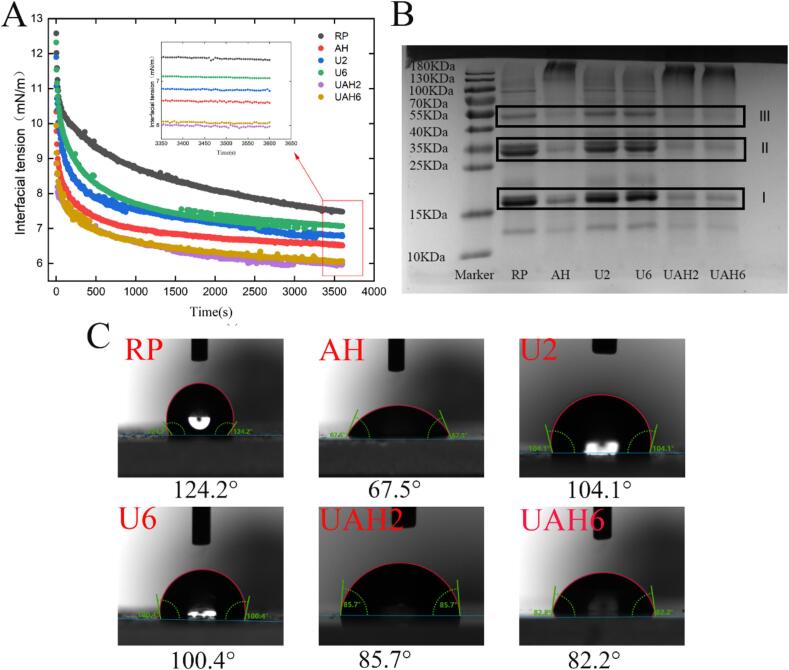
(A) Effects of different modification methods on the interfacial tension of rice protein. (B) Effects of different modification methods on the contact Angle of rice protein. (C) Gel electrophoresis profiles of different modified rice proteins. Note: Rice protein (RP), Alkali-thermal treatment (AH), 200 W ultrasonic treatment (U2), 600 W ultrasonic treatment (U6), 200 W ultrasonic-alkali-thermal treatment (UAH2), 600 W ultrasonic-alkali-thermal treatment (UAH6).

The adsorption behavior of proteins at the oil–water interface reflects their ability to stabilize emulsions[35]. The greater the interfacial tension at the oil–water interface, the less energy is required to disrupt the emulsion interface, leading to poorer emulsion stability[36]. As shown in Fig. 3A, the interfacial tensions of UAH2 and UAH6 are 5.96 ± 0.48 mN·m^−1^ and 6.05 ± 0.52 mN·m^−1^, respectively, which are much lower than that of RP (7.48 ± 0.79 mN·m^−1^). This may be attributed to the exposure of hydrophilic groups after ultrasonic-alkali-thermal treatment, enhancing the protein's binding capacity at the water interface and significantly reducing the interfacial tension. The contact angle results (Fig. 3C) also illustrate this phenomenon. The contact angle of RP is 124.2°, whereas after ultrasonic-alkali-thermal modification, the contact angles of UAH2 and UAH6 are 85.7° and 82.2°, respectively. This indicates that ultrasonic-alkali-thermal treatment can significantly enhance the hydrophilicity of the protein, reducing its contact angle closer to 90° and thereby imparting better intermediate wettability to the protein. The results of the interfacial adsorption characteristics suggest that UAH2 and UAH6 may be more suitable as stabilizing agents for emulsions.

### 3.9 SDS-PAGE

Gel electrophoresis was used to analyze the molecular weight and structural changes of rice protein before and after modification. As shown in Fig. 3B, the bands of U2 and U6 do not change significantly compared to RP, indicating that ultrasonic treatment does not cause changes in the molecular weight of rice protein. Meng et al. [37]also found that ultrasonic treatment does not change the molecular weight of whey protein. When comparing AH, UAH2, and UAH6 with RP, it was found that the subunits I, II, and III of heat-treated rice protein all showed band fading, with bands gathering at the top of the gel. This is because heat treatment causes depolymerization of low-molecular-weight subunits, which then undergo thermal aggregation to form large-molecular-weight protein subunits. Since the separation gel only allows molecules with a molecular weight less than 180 kDa to pass through, molecules larger than this weight accumulate at the top of the gel [38].

### 3.10 Analysis of the apparent morphology of proteins

**Fig. 4:**
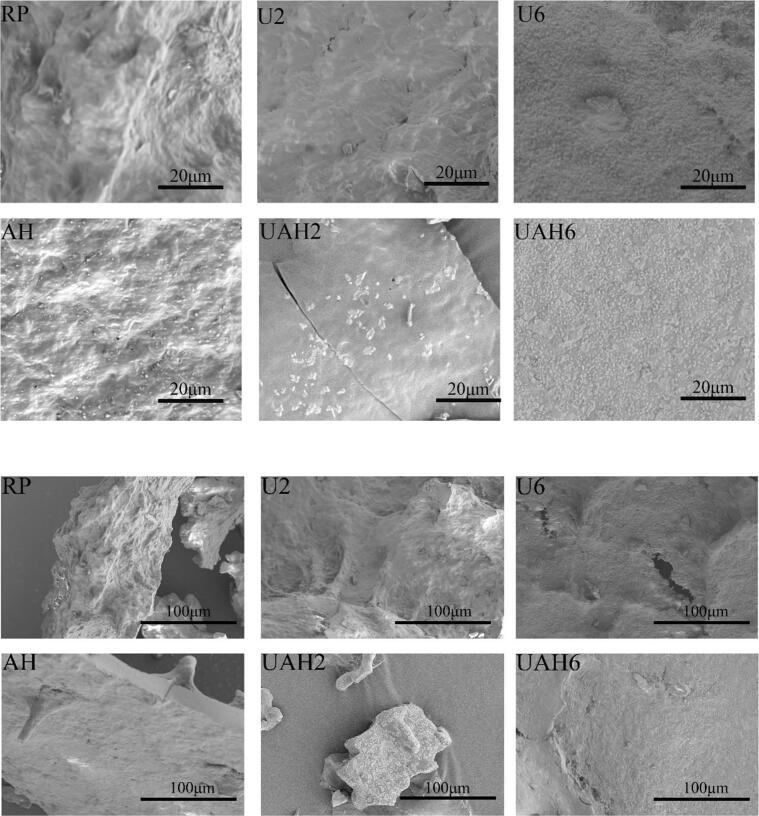
SEM images of different modified rice proteins. Note: Rice protein (RP), Alkali-thermal treatment (AH), 200 W ultrasonic treatment (U2), 600 W ultrasonic treatment (U6), 200 W ultrasonic-alkali-thermal treatment (UAH2), 600 W ultrasonic-alkali-thermal treatment (UAH6).

The apparent morphology of rice protein before and after modification was observed by SEM. As shown in Fig. 4, the sample surface of RP is rough and has many irregular shallow pits. After ultrasonic treatment, it can be observed that the shallow pits on the surface of the U2 sample disappear and numerous minute fissures emerge. flake-like protrusions appeared on the surface of the U6 sample. After heat treatment, the surfaces of the AH, UAH2, and UAH6 samples became smoother compared to those that had not undergone heat treatment. These results indicate that protein structure changes after modification, and these findings are consistent with the results of ultraviolet spectroscopy and secondary structure analysis.

### 3.11 Potential and particle size of high-internal-phase emulsions

**Fig. 5:**
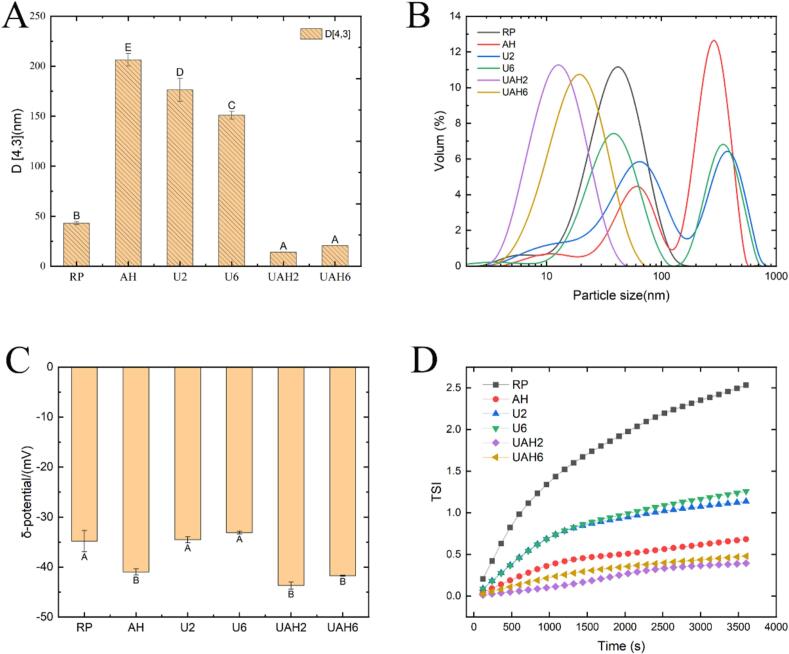
(A) Effects of different modification methods on the particle sizes D43 of rice protein-stabilized emulsions. (B) Effects of different modification methods on the particle size distribution of rice protein-stabilized emulsions. (C) Effects of different modification methods on the emulsion potential of rice protein-stabilized emulsions. (D) Changes in TSI values of rice protein-stabilized emulsions within 1 h by different modification methods. Note: Different letters between the results denote statistically significant differences (p < 0.05). Rice protein (RP), Alkali-thermal treatment (AH), 200 W ultrasonic treatment (U2), 600 W ultrasonic treatment (U6), 200 W ultrasonic-alkali-thermal treatment (UAH2), 600 W ultrasonic-alkali-thermal treatment (UAH6).

D_43_ represents the average value of droplet volume distribution. A smaller D_43_ value indicates a more uniform droplet distribution in the emulsion and fewer larger droplets [39]. The D_43_ values and particle size distributions of the emulsions are shown in Fig. 5A and 5B. Both UAH2 and UAH6 exhibited unimodal particle size distributions, with D_43_ values of 14.17 ± 0.01 μm and 20.57 ± 0.01 μm, respectively. This demonstrates that these two emulsions had relatively uniform particle size distributions. The other four emulsions all showed bimodal distributions and larger D_43_ values, indicating non-uniform particle size distributions with significant presence of larger droplets. The difference between the two was also much greater than that of the above two groups, indicating that the particle size distribution of the emulsions in these four groups was uneven and there were large droplets. The particle size results of the emulsion are consistent with those of confocal microscopy and optical microscopy. The potential of the emulsion can be used to evaluate the stability of the emulsion system. A higher absolute potential value indicates that there is a large electrostatic repulsion between the emulsion droplets, which can prevent the mutual aggregation of the droplets and contribute to the stability of the emulsion system. As can be seen from Fig. 5C, the stable emulsions of the heat-treated proteins (AH, UAH2, UAH6) have a relatively high absolute potential. This might be due to the heat treatment causing the disulfide bonds of the rice protein to break, exposing the originally folded polar amino acid residues inside [40]. The result of emulsion potential is consistent with the stability result.

### 3.12 Storage stability analysis

**Fig. 6:**
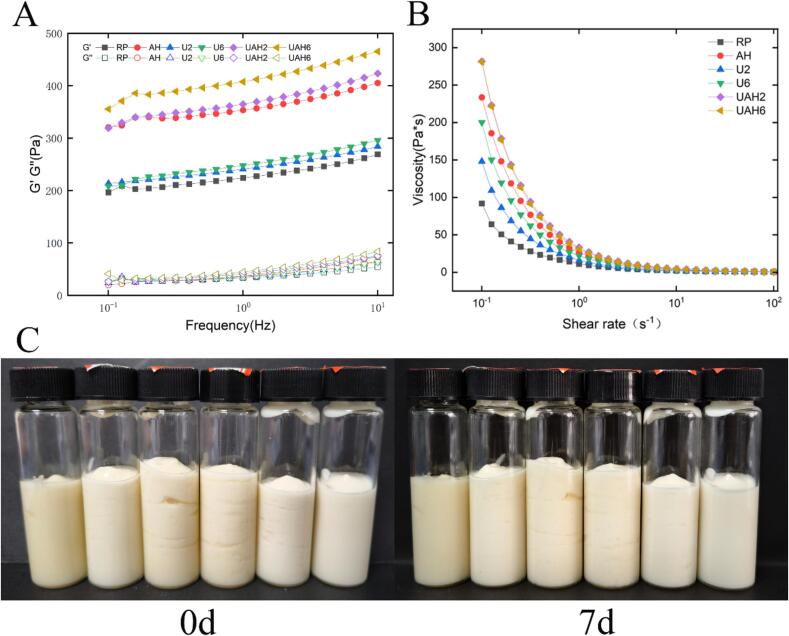
(A) Effects of different modification methods on the energy storage modulus and loss modulus of rice protein-stabilized emulsions. (B) Effects of different modification methods on the apparent viscosity of rice protein-stabilized emulsions. (C) Macroscopic static images of rice protein-stabilized emulsions at 0 days and 7 days (from left to right: RP, AH, U2, U6, UAH2, UAH) 6). Note: Rice protein (RP), Alkali-thermal treatment (AH), 200 W ultrasonic treatment (U2), 600 W ultrasonic treatment (U6), 200 W ultrasonic-alkali-thermal treatment (UAH2), 600 W ultrasonic-alkali-thermal treatment (UAH6).

TSI is a key indicator used to evaluate emulsion stability and is determined using Turbiscan multiple light scattering technology. The higher the TSI value and the greater the slope, the more significant the tendency of phase separation (e.g., stratification, sedimentation, or floating) during storage or under external forces, indicating poorer emulsion stability. Conversely, lower TSI values and shallower slopes indicate better emulsion stability[41]. As shown in Fig. 5D, RP has the highest TSI value of 2.53. This may be due to the larger particle size of unmodified protein (Fig. 1B), which results in larger emulsion droplets and poorer stability. The TSI values of protein-stabilized emulsions decreased to varying degrees after treatment. Among these, UAH2 had the lowest TSI value of 0.39, followed by UAH6 at 0.48. The modified protein had a smaller particle size (Fig. 1B), resulting in smaller, more uniform emulsion droplets and better stability. The results show that ultrasonic-alkali-thermal treatment reduced the TSI values of protein-stabilized emulsions to relatively low levels, significantly improving their stability. The macroscopic standing image of the emulsion (Fig. 6C) shows that freshly prepared UAH2 and UAH6 emulsions are snow-white, indicating complete protein encapsulation of oil droplets. The other four groups appear yellowish, indicating the presence of unencapsulated oil droplets. After seven days, UAH2 and UAH6 emulsions showed no significant changes, whereas the other four groups exhibited slight oil separation at the top. These results are consistent with other emulsion data.

### 3.13 Rheological property analysis

The rheological properties of emulsions mainly include two aspects: apparent viscosity and viscoelasticity. Higher apparent viscosity can prevent droplet collision and aggregation, thereby improving emulsion stability [42]. As shown in Fig. 6B, the apparent viscosity of all HIPEs decreases with increasing shear rate, indicating that the emulsion exhibits shear-thinning behavior and is a pseudoplastic fluid[43]. UAH2 and UAH6 show relatively high apparent viscosity. This may be due to the smaller particle size and closer packing of emulsion droplets in these two groups, which increases apparent viscosity. This result is consistent with the emulsion particle size results.

The viscoelasticity of an emulsion is primarily characterized by its moduli. The energy storage modulus (G') reflects the elasticity of the emulsion and indicates its solid-like behavior. The loss modulus (G'') reflects the viscosity of the emulsion and indicates its liquid-like behavior [38]. Fig. 6A shows the variation curves of G' and G'' for different HIPEs as a function of scanning frequency. It can be seen that G' is greater than G'' for all emulsions, indicating their predominantly elastic nature and solid-like properties. The G' values of UAH6 and UAH2 were higher than those of the other treatment groups and the untreated group, indicating that the gel structures formed by these two groups were more compact and exhibited better anti-deformation ability. This may be because the emulsion stabilized by rice protein after ultrasound-alkali-heat treatment has a smaller particle size, and the closer packing of droplets enhances viscoelasticity. The rheological results of the emulsion are consistent with the emulsion particle size results.

### 3.14 Microstructure

**Fig. 7:**
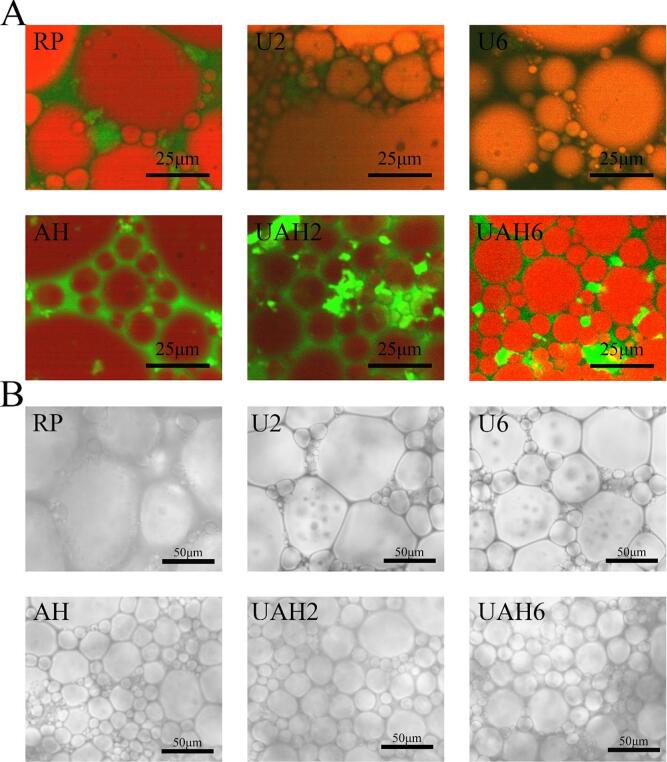
(A) Emulsion laser confocal images of rice protein stabilization by different modification methods. (B) Emulsion optical microscope images of rice protein stabilization by different modification methods. Note: Rice protein (RP), Alkali-thermal treatment (AH), 200 W ultrasonic treatment (U2), 600 W ultrasonic treatment (U6), 200 W ultrasonic-alkali-thermal treatment (UAH2), 600 W ultrasonic-alkali-thermal treatment (UAH6).

Fig. 7A shows the laser confocal microscope image of the emulsion. It can be clearly observed that the red fluorescent oil droplets are wrapped by the green fluorescent protein, and the droplets are closely stacked with each other. This is a typical feature of a high-internal-phase emulsion, indicating that this emulsion is an O/W type high-internal-phase emulsion. The green fluorescence fills the gaps between the droplets, indicating that the protein effectively adsorbs on the surface of the oil droplets to form a tight interfacial layer, which can effectively prevent collisions between small droplets. The emulsion droplets of UAH2 and UAH6 are the smallest and most evenly distributed, which can effectively prevent aggregation. In contrast, AH contains many large droplets and only a few small droplets. The emulsion droplets of U2 and U6 are unevenly distributed and irregular in shape. In the emulsion droplets of RP, a relatively large proportion are large droplets. Large droplet sizes or uneven distribution can lead to emulsion aggregation, causing the particle size of the emulsion to increase or demulsification, and making it unstable during storage. Fig. 7B shows the optical microscope results. The image results are consistent with the laser confocal results. The droplets are closely packed and piled up with each other, presenting the characteristics of a high-internal-phase emulsion. The microstructure is consistent with the particle size results.

### 3.15 Mechanism analysis of Ultrasonically – Alkali-Thermal treatment on rice protein and its stabilization of emulsions

Under alkaline conditions (pH 12), the compact structure of rice protein unfolds, whereupon ultrasonic treatment flips the exposed hydrophobic groups containing phenylalanine residues inward while exposing weakly hydrophobic groups with tyrosine residues; finally, through thermal treatment, the rice protein transitions into a molten globule state wherein phenylalanine-embedded hydrophobic groups become buried deeper (evidenced by decreased UV absorbance) and tyrosine-containing weakly hydrophobic groups remain exposed (evidenced by increased fluorescence intensity), concurrently, the thermal treatment enhances molecular flexibility of rice protein, facilitating its adsorption at the interface. Concurrently, thermal treatment enhances the molecular flexibility of rice protein, facilitating its adsorption at the interface; consequently, the ultrasonic-alkali-thermal treatment rice protein exhibits improved amphiphilicity (Fig. 3C), enabling rapid adsorption at the oil–water interface during high-internal-phase emulsion formation to establish a dense continuous phase wherein O/W droplets undergo ordered alignment, forming an interconnected three-dimensional gel network through mutual stacking into polyhedral configurations that maintain system stability (Fig. 7), with the resultant emulsion demonstrating elevated elastic modulus and viscosity (Fig. 6) that effectively prevent droplet collision, aggregation, and phase separation.

## 4 Conclusion

The solubility of rice protein after ultrasonic-alkali-thermal treatment has been significantly improved compared with the unmodified protein. The solubilities of UAH2 and UAH6 have increased by 545 % and 631 % respectively, the ESI value has risen, and the emulsification property has improved. The structural modifications induced by ultrasonic-alkali-thermal treatment, particularly in primary, secondary, and tertiary configurations, led to a balanced hydrophilicity-hydrophobicity ratio. The oil–water interfacial tension decreased from 7.48 mN·m^−1^ to 5.96 mN·m^−1^, and the contact angle was close to 90°. The high-internal-phase emulsion prepared based on ultrasonic-alkali-thermal treatment of rice protein has a smaller particle size and a uniform distribution, and has better anti-deformation ability. The TSI value of the emulsion decreased from 2.53 to 0.39, which indicates that the protein-stabilized emulsion modified by ultrasonic-alkali-thermal has better stability. Therefore, ultrasonic-alkali-thermal modification can significantly enhance the emulsification property of rice protein and is a treatment method that can improve the functional properties of rice protein. This modification approach can also be adapted for grain proteins sharing structural similarities with rice protein. Furthermore, the high-internal-phase emulsions investigated in this study warrant further exploration for potential applications, including the delivery of lipophilic substances, customized 3D-printed foods, and fat substitutes.

## CRediT authorship contribution statement

**Lijie Zhu:** Writing – original draft, Investigation, Funding acquisition. **Haiqiang Liao:** Investigation, Data curation. **Kun Zhuang:** Writing – review & editing, Funding acquisition. **Shangyuan Sang:** Writing – review & editing. **Lei Chen:** Software. **Xianhui Chang:** Data curation. **Qi Zhang:** Formal analysis. **Qingyun Lv:** Writing – review & editing. **Xiuying Liu:** Writing – review & editing, Conceptualization. **Xinqi Liu:** Writing – review & editing, Project administration. **Wenping Ding:** Writing – review & editing, Conceptualization.

## Declaration of competing interest

The authors declare that they have no known competing financial interests or personal relationships that could have appeared to influence the work reported in this paper.

## Data Availability

Data will be made available on request.
